# Editorial: Cellular, molecular and immunological aspects in arboviruses infection

**DOI:** 10.3389/fcimb.2022.973953

**Published:** 2022-07-14

**Authors:** Moises Leon Juarez, Julio García-Cordero, Mauricio Comas-Garcia, Leticia Cedillo- Barrón, José González-Santamaría, Gaurav Shrivastava

**Affiliations:** ^1^ Departamento de Inmunobioquímica, Instituto Nacional de Perinatología Isidro Espinosa de los Reyes, Ciudad de México, Mexico; ^2^ Departamento de Biomedicina Molecular, Centro de Investigación y de Estudios Avanzados del Instituto Politécnico Nacional (CINVESTAV-IPN), Ciudad de México, Mexico; ^3^ Sección de Microscopía de Alta Resolución, Centro de Investigación en Ciencias de la Salud y Biomedicina, Universidad Autónoma de San Luis Potosí, San Luis Potosí, Mexico; ^4^ Facultad de Ciencias, Universidad Autónoma de San Luis Potosí, San Luis Potosí, Mexico; ^5^ Grupo de Biología Celular y Molecular de Arbovirus, Instituto Conmemorativo Gorgas de Estudios de salud, Panamá, Panama; ^6^ Laboratory of Malaria and Vector Research, National Institute of Allergy and Infectious Diseases, National Institutes of Health, Rockville, MD, United States

**Keywords:** Flavivirus, Alphavirus, vector, cellular factor, immune responses

## Introduction

Emerging and re-emerging arthropod-borne viruses are collectively referred to as arboviruses (Young, 2018). The global importance of infections caused by these pathogens affects health systems and control of zoonotic diseases, thus promoting a relevant problem in human and animal health worldwide (Chala and Hamde, 2021). Reports generated by the World Health Organization have identified that 17% of infectious diseases are associated with arboviruses and one million deaths annually worldwide. Traditionally, arbovirus research has been of low priority. However, the unprecedented emergence of epidemic arboviral diseases overturns the perception about their impact on global mortality and disability over the past 50 years (Gubler and Clark, 1995; GBD 2013 DALYs and HALE Collaborators et al., 2015). For transmission to occur, these pathogens require arthropod vectors such as mosquitoes, ticks, and sandflies; where the virus is replicated, without any apparent harmful effects on the vector, before being transmitted to their vertebrate host (Oliveira et al., 2020). Additionally, some arboviruses have a series of sylvatic and urban infection cycles. Sylvatic cycle is common to most of arboviruses, where arbovirus amplification occurs in animals that serve as reservoir. Urban infectious cycle is specific to some arboviruses, where they can be transmitted to host such as humans and thereby promote epidemic outbreaks (Higuera and Ramírez, 2019). The arboviruses that infect humans and animals can be concentrated in four families: *Togaviridae*, *Flaviviridae*, *Bunyaviridae*, and *Reoviridae*. It is important to remember that these families are not genetically related, but it is their transmission cycle that groups them as arboviruses. These viruses have been related to a series of human diseases, ranging mainly from hemorrhagic fevers, encephalitis and an arthritis syndrome (Segura et al., 2021). However, the evolutionary changes of several arboviruses have generated a diversification in the tropism and transmission of these pathogens promoting complications during pregnancy or being considered the cause of sexually transmitted infections (Arévalo Romero et al., 2019). The lack of antiviral treatments and the development of effective vaccines that protect against infections by these pathogens urge more efficient efforts to understand and identify the mechanisms involved in different events within the infection cycle of these pathogens, thus defining the vital importance of specific research area. Finally, this Research Topic´s purpose is to generate a series of compelling research articles and a comprehensive review to better understand the host response to arbovirus infections.

An important topic in virology is the connection between cellular metabolism and the crosstalk with viruses, as several viruses have shown to cause metabolic modification to facilitate their productive infection cycle (Sánchez-García et al., 2021). Here, Wald et al. evaluated the impact of glycolysis in the Usutu virus cycle. Interestingly, Usutu virus (*Flaviviridae*) infection increases glycolytic activity, thereby supporting its infection. Furthermore, an interferon response was counteracted by glycolysis stimulated by Usutu virus infection, suggesting a connected regulatory pathway for innate resistance and metabolism. The authors concluded that glycolysis is a key target for therapeutic intervention against Usutu infection and emphasizes the importance of the development of viral antagonists of the IFN pathway. Continuing with the same theme, an organelle that has implications in cellular bioenergetics and metabolism is the mitochondria. The relationship between viruses and mitochondria is a recent topic that involves different mechanisms to control or benefit viral infection. Here, Rodriguez et al. observed the effect of the Sindbis virus (*Togaviridae*) in the host mitochondria and demonstrate the reduction of mitochondrial staining intensity, ATP production, and oxygen consumption These findings suggest that the Sindbis virus uses alternative non-mitochondrial metabolic pathways in the early phases of viral replication.

A critical point of infection with arboviruses is the phenotypic variability of these viruses when isolated from epidemic outbreaks and how these can have a differential impact on the host’s immune response. For example, Castro-Jimenez et al. Performed a comparative analysis of replication kinetics between different clinic isolates of Dengue and Zika viruses (*Flaviviridae*) obtained from blood samples of febrile patients in the Oaxaca region of México. This study demonstrates the correlation between interferon susceptibility and subgenomic RNA accumulation, with clinical manifestations such as high hematocrit percentage and thrombocytopenia. The development of antiviral strategies for arbovirus infections is a topic of vital importance. Several plant extracts have been exploited in this context to make synthetic drugs with superior activity and reduced toxicity with potential antiviral activity against some arboviruses (Dhawan, 2012). Here, Alagarasu et al. analyzed 25 plant extracts to perform an antiviral screening for the Dengue and Chikungunya (*Togaviridae*) infection. Their results showed that four of the plant extracts generated an inhibition of the infection in parallel with the two viruses evaluated, but only one showed a virus-specific inhibitory activity. These findings highlight the characterization of compounds isolated from these plants that can function as effective phytopharmaceuticals against some arboviruses.

Finally, an excellent review was included in this Research Topic. Here, Luria-Perez et al. discussed the implementation of an alternative route for applying vaccines against flaviviruses. During their description. The authors explain the different platforms used to develop vaccines against flaviviruses and proposed under-explored strategies for their administration. They consider the mucosal route as a novel and beneficial strategy to deliver antigens and promote more efficient stimulation of the immune system, supporting the idea of its implementation for control of arbovirus infection.

The global impact of arbovirus infections is associated with climatic changes that promote a more significant extension of the habitat of vectors, making the diseases related to these viruses relevant in public health. Therefore, the research focused on understanding the interaction of the host with the viruses and its relationship with the pathogenic mechanisms is imperative to generate strategies for the control and development of a solution for its implementation in the clinic. In addition, the recent outbreaks of emerging flaviviruses underline the need to better understand these viruses. While it was hard to predict which flavivirus will evolve to present global health issues, their changing epidemiology raises concern for large-scale emergence and disease. Therefore, sustained research efforts on arboviruses are needed.

In conclusion, we thank the authors and reviewers who participated in the generation of these puzzling pieces in generating knowledge about arboviruses. This Research Topic is timely, and we hope that it will serve as an inspiration for future research that identifies new mechanisms that help develop future strategies to control arbovirus infections.

## Author contributions

ML-J and GS wrote the first draft of the manuscript. JG-C, MC-G, LC-B and JG-S revised the manuscript. All authors contributed to the article and approved the submitted version.

## Conflict of interest

The authors declare that the research was conducted in the absence of any commercial or financial relationships that could be constructed as a potential conflict of interest.

## Publisher’s note

All claims expressed in this article are solely those of the authors and do not necessarily represent those of their affiliated organizations, or those of the publisher, the editors and the reviewers. Any product that may be evaluated in this article, or claim that may be made by its manufacturer, is not guaranteed or endorsed by the publisher.
